# Managing COVID-19 related distress in primary care: principles of assessment and management

**DOI:** 10.1186/s12875-021-01399-8

**Published:** 2021-04-14

**Authors:** Laurence Astill Wright, Sam Gnanapragasam, Anthony J. Downes, Jonathan I. Bisson

**Affiliations:** 1 Division of Psychological Medicine and Clinical Neurosciences, Cardiff University School of Medicine, Cardiff, UK; 2 The Institute of Psychiatry, Psychology and Neuroscience, King’s College London, London, UK

**Keywords:** Psychological Distress, Traumatic Stress Symptoms, Anxiety, Depression, PTSD, COVID-19

## Abstract

COVID-19 will cause normal feelings of worry and stress and many of those who experience higher levels of distress will experience resolution of their symptoms as society returns to pre-COVID-19 functioning. Only a minority are likely to develop a psychiatric disorder. Certain individuals may be vulnerable to experiencing persisting symptoms, such as those with pre-existing comorbidity. Management approaches could centre around using collaborative approaches to provide and build on already existing socioeconomic support structures, the avoidance of over-medicalisation, watchful waiting and finally treating those who do meet the criteria for psychiatric diagnosis. Primary care clinicians are likely be the first healthcare point of contact for most COVID-19 related distress and it is important that they are able to provide evidence based and evidence informed responses, which includes social, psychological and pharmacological approaches. This expert opinion paper serves to summarise some approaches, based primarily on indirect extrapolation of evidence concerning the general management of psychological distress, in the absence of COVID-19 specific evidence, to assist primary care clinicians in their assessment and management of COVID-19 related distress.

The psychological consequences of COVID-19 are likely to be broad and affect millions of people worldwide. COVID-19 may cause psychological and emotional distress, commonly manifesting in grief and in symptoms of anxiety, depression and traumatic stress, much of which will represent a normal reaction to an abnormal situation and subside without the need for formal intervention. Primary care clinicians are likely to be the first healthcare point of contact for most COVID-19 related distress and it is important that they are able to provide evidence-informed responses, which includes social, psychological and pharmacological approaches. This paper, which is based on a non-systematic review of the literature and expert opinion, aims to explore and predict patterns of psychological distress during the COVID-19 pandemic, including when and what interventions to offer in primary care.

## Stressors, manifestations and course of distress

**Fig. 1:**
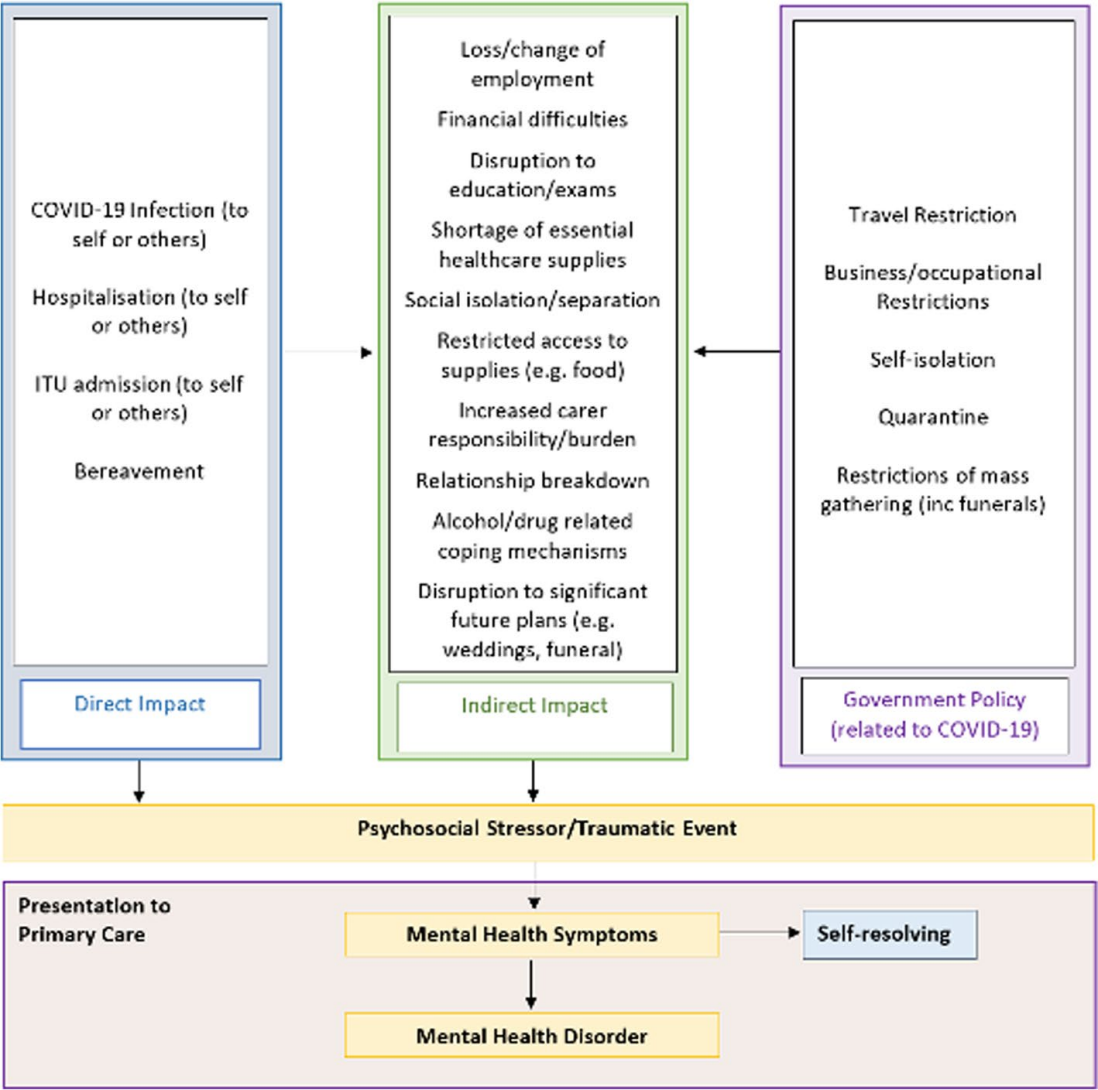
COVID-19 related direct and indirect causes and symptoms of psychological distress

The trajectory of COVID-19 and magnitude of its impact on life across the world, suggest that the psychological challenges may differ from previous pandemics in scale and possibly also in nature via multiple psychosocial stressors and secondary socioeconomic consequences. COVID-19 may affect individuals in a variety of ways with direct effects on individuals (e.g., via hospitalisation and bereavement) and indirect effects (e.g., social isolation, financial hardship), which are compounded by shifting government policies (Fig. 1). As such, it is likely that COVID-19 will have a profound impact on the social determinants of health, including disruptions to healthcare provision, education, economic stability, community networks and accessibility to physical environment.

Some of these direct life events will meet the traumatic stressor criterion for Diagnostic and Statistical Manual of Mental Disorders Fifth Edition (DSM-5) Post traumatic Stress Disorder (PTSD), such as experiencing or witnessing actual or threatened death. However, many indirect stressors will not lead to PTSD but might still cause distress. Stressful life events are one of the main precipitating factors for psychopathology and COVID-19 related psychosocial stressors will likely precipitate a range of psychological reactions, including mental disorder.

It is common and normal for individuals to feel stressed during a time of such significant global upheaval (Fig. 1). For most people, this initial psychological upset will gradually decrease as the external stressor subsides. The majority of the general population will show good adaptability and resilience to psychosocial stressors, and some individuals may even have positive experiences (e.g. pride in their ability to cope in crisis ([1] and reduced anxiety amongst some adolescents [2]). Only a minority of individuals are likely to experience pathological distress which will probably be characterised as symptoms of anxiety, depression, grief, traumatic stress and maladaptive coping behaviours such as substance misuse [3].

These predictions are supported by the emerging evidence. Recent systematic reviews and meta-analyses demonstrate wide ranging prevalence rates of symptoms of anxiety, depression and traumatic stress [4–6]. Peer reviewed studies of higher methodological quality demonstrated lower prevalence rates of posttraumatic stress (17.38% in published studies) compared with those of lower methodological quality (34.71% in unpublished studies) [4]. In the United Kingdom, a study of 2000 adults representative of the British population 52 days after the first confirmed case of COVID-19 demonstrated only slightly higher rates of traumatic stress (female: 14.9%, male: 18.9%), anxiety (female: 25.1%, male 17.9%) and depression (female: 23.4%, male 20.6%) compared to pre-COVID-19 UK population estimates [3]. This highlights the remarkable psychosocial resilience of the majority of the general population. While direct comparisons are not possible due to methodological differences, these findings are broadly similar to the prevalence of distress experienced during SARS-CoV-1 [7, 8].

Studies examining the psychological consequences of other pandemics [9] suggest that some non-specific symptoms of emotional distress (which have been characterised as adjustment disorder—psychological distress in response to a significant life event – by some authors) will be just as common as those of PTSD [10]. COVID-19 related mortality will also leave many people bereaved, experiencing entirely normal but highly distressing symptoms related to the anguish of losing someone close, such as anger, guilt, regret and loneliness. Furthermore, the cultural norms that support a normal grieving process will be disrupted (e.g., inability to attend their funeral), increasing the likelihood of pathological outcomes such as prolonged grief disorder. Loss of social support due to bereavement or unemployment, could add to the risk of individuals developing psychiatric problems and maladaptive coping strategies such as substance abuse.

Similar to normal feelings of worry and stress related to COVID-19, many of those who experience higher levels of distress will experience resolution of their symptoms as society gradually returns to pre-COVID-19 functioning. Only a minority may develop a psychiatric disorder, such as anxiety, depression, adjustment disorder, prolonged grief disorder and PTSD [11]. Relatively few studies have assessed the rate of formal psychiatric disorder in the general population following epidemics, with most using assessment measures insufficient to diagnose [12] and not assessing pre-existing psychiatric comorbidity [11].

## At risk population groups

Prevalence studies of hospitalised SARS patients have demonstrated a psychiatric disorder prevalence of 42.5% in 233 individuals up to 4 years post-infection, albeit in a particularly high risk group [13]. Those requiring higher levels of medical input for COVID-19 related illness will be more likely to develop psychiatric conditions requiring treatment, as observed following the 2009 H1N1 pandemic [14]. Estimates of total numbers of patients requiring critical care vary markedly, but of confirmed cases of COVID-19, however, 15% will require hospitalisation and 5% will require invasive mechanical ventilation on an Intensive Therapy Unit [15]. The prevalence of PTSD symptoms following ITU discharge is 24% at 6 months and 22% at 12 months [16, 17], comparable to that of military combat and major physical injury [18]. Analysis of patients treated for SARS-CoV-1 highlights that greater perceived life threats are associated with worse PTSD symptom severity [19]. This imminent threat to life by an entirely novel and poorly understood virus may be combined with worry of spreading the contagious virus to vulnerable friends and family.

COVID-19 related distress will likely disproportionally affect those with more traumatic COVID-19 exposure and other more vulnerable groups, including those with pre-existing psychiatric and physical health comorbidity [3]. As most people with pre-existing comorbidities are managed within community settings this will pose particular challenges to primary care services. Multiple shared vulnerability factors, such as adverse childhood experiences and low socioeconomic status, are common trans-diagnostically with pre-existing psychiatric disorder likely to increase susceptibility to other psychiatric conditions [20]. Furthermore, COVID-19 may exacerbate pre-existing socioeconomic vulnerability and economic recessions are associated with increased prevalence of psychiatric morbidity [21].

Healthcare workers who are predominantly female, may be particularly vulnerable [22], with healthcare workers with SARS demonstrating poor emotional adjustment following physical illness [12]. Clear communication and support within the workplace clearly affects subsequent distress, [23] and while individuals may not initially appreciate the severity of their distressing experiences at the time, employers should consider the mental health consequences of inadequate support structures [24]. Other occupations with high viral exposure requiring institutional support include supermarket workers.

Early evidence suggests that there is a higher COVID-19 related psychological distress prevalence in women, who are also more likely to be bereaved [25, 26]. A multitude of social and biological differences explain the higher rates of PTSD in women regardless of trauma type [27]. Women tend to seek more social support than men, and isolation and quarantine will decrease this available social support, a lack of which is the most consistent correlate of poor outcomes after trauma [27]. Early reports of increased call volumes to the National Domestic Abuse Helpline [28] further highlight women’s potential vulnerability.

Lack of social support is likely to exacerbate distress related with both COVID-19-related and non-COVID-19-related traumatic events. While isolation and quarantine does not meet the PTSD stressor criterion, some individuals may feel the experience overwhelms their psychological ability to cope. The psychological impacts of quarantine and isolation are broad and highlight the effect of isolation greater than 10 days in increasing traumatic stress symptoms, anger and avoidance [12]. Interestingly, early research suggests that people over 65 report lower rates of COVID-19 related anxiety and depression compared to younger people, suggesting greater psychosocial resilience despite higher risk of serious illness [3, 29].

## Principles of intervention & treatment approaches

**Fig. 2:**
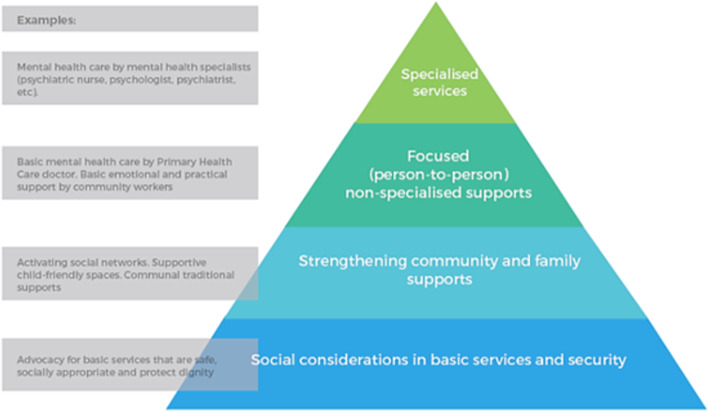
Intervention pyramid for mental health and psychosocial support (Reproduced from IASC 2020—permission has been requested to reproduce this figure)

There remains considerable uncertainty in how best to manage the psychosocial consequences of COVID-19. This paper summarises the approaches recommended by a variety of organisations [1, 30], based primarily on the indirect extrapolation of evidence concerning the general management of psychological distress, in the absence of COVID-19 specific evidence. The large heterogeneity in demographics affected by COVID-19 will cause varied manifestations of psychological distress requiring a range of approaches (Fig. 2). General overarching principles are centred around the avoidance of over-medicalisation and further harm, using collaborative approaches to provide and build on already existing socioeconomic support structures, watchful waiting and finally treating those who do meet the criteria for psychiatric diagnosis [1]. Stepped care interventions should be multi-faceted and function within an integrated support system of multiple coordinating groups acting in response to COVID-19 [1].

Initially, individuals will require easy access to basic services providing practical and financial security to protect their health and wellbeing. These services should be safe, socially appropriate, preserve the dignity of those accessing them and be able to adapt to changing needs [1], e.g. ensuring vulnerable groups still retain access to shelter, care and medication. Following this, the COVID-19 response should seek to enable and extend existing family and community support structures through providing COVID-19 specific knowledge, teaching new supportive skills (such as psychological first aid) and increasing awareness of the care pathways in place for distressed individuals to access specialist support [1]. Employers of staff vulnerable to psychological distress should implement clear guidance around policies, practices and occupational support structures [31] and avoid stigmatising those with COVID-19 or related distress [30].

For those individuals displaying heightened distress, primary care practitioners can offer emotional, practical and pragmatic support. This serves to cope with worries arising directly from COVID-19 (e.g., exposure to the disease and survivors’ stories of suffering, loneliness and powerlessness) and those indirectly related (e.g., an inability to engage in recreation, poor organisation and occupational role definitions) [31]. Individuals should be encouraged to maintain the social support structures and coping strategies they already possess within the confines of social distancing [30] but there is no one size fits all solution to the psychosocial requirements of an entire society [1]. Coping strategies may include self-care techniques, such as relaxation and cognitive exercises, physical exercise and providing support on how to access reliable information about COVID-19 related developments.

## COVID-19 related mental illness

The ability to distinguish between a normal reaction to external stressors and a pathological one will be key for primary care practitioners, who may already struggle to differentiate between psychological distress and mental disorder due to contrasting frameworks around the conceptualisation of each [32]. This may be particularly challenging acutely, before the possibility of longitudinal assessment and before any basic supportive interventions have been offered [33]. Pathological reactions typically involve a significant impact on an individual’s functioning with a higher severity of symptoms persisting over a prolonged period of time and often beyond the resolution of a precipitating psychosocial stressor. Pathological reactions may be associated with higher degrees of risk, the management of which should be a key consideration during initial consultation. Assessment should appreciate that while direct COVID-19 stressors may resolve first, for many, the indirect impact of the pandemic, such as financial hardship, will persist for longer.

For the small minority of individuals who develop signs and symptoms of mental disorder and for whom basic emotional and pragmatic support is insufficient, evidence-based psychological and/or pharmacological treatments should be offered based on the primary presenting diagnosis (e.g. Depression [33], PTSD [34]). Many of these interventions will be provided in primary care settings but some individuals will have more complex difficulties or risks, including suicide risk, that require involvement of secondary care services.

## Limitations

This paper, which is not the result of a systematic literature search, is based on a non-systematic review of the literature and our knowledge of the area, serves to summarise some of the available evidence to assist primary care clinicians in their assessment and management of COVID-19 related distress. Consistent with a recently published rapid systematic review of the literature [35], most of the studies considered have limited internal and external validity and a high risk of selection bias (small sample sizes from single geographical regions with short follow up periods). Consequently, our recommendations should be employed judiciously and always in a personalised manner that integrates clinical expertise, individual patients’ values and preferences, and the available evidence.

## Conclusions

COVID-19 related distress may manifest in symptoms of depression, anxiety, grief and traumatic stress, the majority of which does not require formal medical treatment. Those particularly vulnerable to persisting symptoms and the development of psychiatric conditions are those with pre-existing conditions, females, health care workers, those with low social support, possibly exacerbated by isolation and quarantine, and those facing indirect socioeconomic consequences such as unemployment. For those who develop mental health disorders, effective evidence-based treatments are available. The psychiatric consequences of the COVID-19 pandemic will likely first present in primary care and primary care clinicians have the potential to mitigate their consequences.

## Data Availability

Not applicable.
